# Supplementary figures and images for: Overwintering Larval Cold Tolerance of Sirex noctilio (Hymenoptera: Siricidae): Geographic Variation in Northeast China
Source: Insects. 2021 Jan 28;12(2):116. doi: 10.3390/insects12020116 (PMC7911264; doi:10.3390/insects12020116)

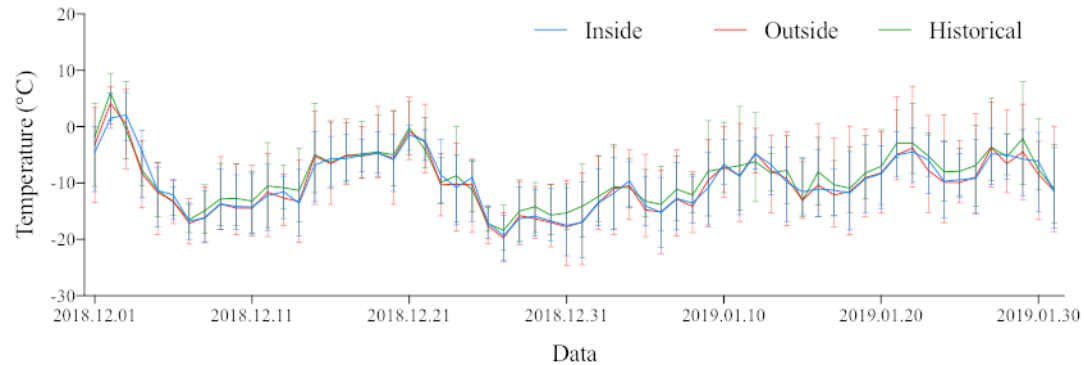

Supplement: Supplementary file 1 [file insects-12-00116-s001.pdf]
